# Bacterial DNA detection in blood using PCR/ESI-MS in neonates with suspected early onset infection

**DOI:** 10.3389/fcimb.2025.1579624

**Published:** 2025-10-21

**Authors:** Ajay K. Sinha, Anja Saso, Arikana Mufunde, Steven T. Kempley, Mark Wilks, Mike Millar

**Affiliations:** ^1^ Neonatal Unit, Royal London Hospital, Barts Health NHS Trust, London, United Kingdom; ^2^ Blizard Institute, Barts and the London School of Medicine and Dentistry, Queen Mary University of London, London, United Kingdom; ^3^ Department of Microbiology, Royal London Hospital, Barts Health NHS Trust, London, United Kingdom

**Keywords:** early onset sepsis (EOS), infection, neonate, microbiology, blood culture, Polymerase Chain Reaction coupled with Electrospray Ionization Mass Spectrometry (PCR/ESI-MS), bacterial DNA

## Abstract

**Objective:**

The study aimed to assess the relationship between clinical features, routine laboratory parameters, including conventional blood culture, and identification of microorganisms by a commercial system of Polymerase Chain Reaction coupled with Electrospray Ionization Mass Spectrometry (PCR/ESI-MS) (Abbot Iridica) in infants with suspected early-onset infection.

**Study design:**

Prospective observational cohort study.

**Setting:**

Neonatal intensive care unit and postnatal ward at a tertiary hospital within an urban setting.

**Patients:**

Neonates >=34 weeks gestation with clinically suspected early-onset infection between January 2016 and March 2017 were recruited. Blood samples were taken at the time of suspected infection for both blood culture inoculation (BacT/ALERT^®^ system) and PCR/ESI-MS analysis (0.5ml). An electronic database was used to document demographic and clinical details.

**Results:**

54 infants were studied with a median (IQR) gestational age and birth weight of 39.7 (37.5-41.0) weeks and 3.2 (2.7-3.5) kg respectively. 1 infant had both bacterial DNA detected on PCR/ESI-MS and bacterial growth on blood culture (Group B *Streptococcus*). 9 infants had bacterial DNA detected but with negative blood culture. The bacteria identified were *Streptococcus* sp *(n=3) Sneathia (n=1), Cutibacterium acnes(n=6)*,. All infants with no bacterial DNA detected on PCR/ESI-MS also had a negative blood culture result. Infants with positive bacterial DNA identification in blood had significantly higher CRP values; initially (p=0.002), when repeated after 18-24 hours (p=0.02) and maximally within the first 72 hours (p=0.03). The proportion of infants with a CRP> 5 mg/L was significantly higher if bacterial DNA had been detected (p=0.01).

**Conclusions:**

PCR/ESI-MS detected bacterial DNA of organisms considered pathogenic in four times more blood samples than culture alone, and had a high sensitivity and negative predictive value. Bacterial DNA was detected by PCR/ESI-MS in infants who did not have bacterial growth on blood culture and this was associated with a raised inflammatory marker. It may be a useful tool to exclude sepsis in the neonatal cohort and reassess the need for prolonged antibiotic treatment. The results are promising but there is a need to improve blood collection methods to take advantage of the potential benefits of molecular detection.

**What is already known on this topic:**

-Blood culture remains an imperfect gold-standard for the diagnosis of sepsis and has disadvantages in the neonatal cohort, including requiring a large blood volume, being time-consuming and often yielding false negative results due to inadequate blood volume, preexisting antimicrobial therapy and fastidious organisms not growing.
-Given the concerns about antimicrobial stewardship and adverse outcomes associated with antibiotic overuse, other diagnostic tools and biomarkers of infection are required.
-Molecular methods have the potential to overcome many limitations of blood culture and are currently under investigation. However, in many recent studies such methods have proven useful rather as an adjuvant not a replacement of blood culture in various patient groups. Several studies have found that although a significantly higher number of potential pathogens can be detected, some culture positive cases may be missed

**What this study adds:**

-PCR/ESI-MS offers rapid identification of micro-organisms within 6-8 hours with a high negative predictive value which may be useful in excluding sepsis in the newborn.
-There is a correlation between bacterial DNA detected by PCR/ESI-MS and raised levels of CRP in neonates treated for early onset sepsis (EOS).
-The detection of potential contaminants remains a challenge with the use of PCR/ESI-MS for neonatal sepsis even while using a higher threshold for the detection of *Cutibacterium* as determined by the manufacturer. The significance of detection of *Cutibacterium* DNA is newborn blood is uncertain.
-PCR/ESI-MS may ensure early reassessment of the need for antibiotics, thereby promoting antimicrobial stewardship and decreasing morbidity associated with prolonged antibiotic therapy.

## 1 Introduction

Neonatal sepsis accounts for high morbidity and mortality in children under 5 worldwide (Liu et al., 2016). Mortality rates even in high-income countries may be as high as 30% despite improvements in neonatal care (Stoll et al., 2011). Prompt diagnosis and treatment of neonatal early-onset sepsis (EOS) are crucial to prevent severe morbidity and mortality and is considered to be an important standard of care (Neonatal infection (early onset): antibiotics for prevention and treatment | Guidance and guidelines, 2012). Up to 7% of term and late-preterm neonates in high-income countries receive antibiotics during the first 3 days of life if they are suspected to have early-onset sepsis (Vergnano et al., 2011). Furthermore, 10% of all live births with clinical problems requiring admission to neonatal units are also evaluated for infection (Russell et al., 2012).

Blood culture is the gold-standard in the microbiological diagnosis of sepsis, although it has several limitations. Only a minority of these infants demonstrate culture-proven sepsis with a prevalence of 0.1% or less in high-income countries (Vergnano et al., 2011; Escobar et al., 2014; Fjalstad et al., 2016; Cailes et al., 2018). This is partly because blood cultures require a substantial volume of blood to reach adequate sensitivity and this is often difficult to obtain, particularly in newborn infants (Kellogg et al., 1997). Furthermore, blood cultures take time to produce a result, often several days after clinical suspicion. Initiation of antibiotic treatment is therefore determined on the basis of risk factors for sepsis and initial clinical signs; moreover, subsequent duration of therapy depends on an evaluation of both the clinical and biochemical response (change in inflammatory markers) to antibiotics as well as blood culture results (Neonatal infection (early onset): antibiotics for prevention and treatment | Guidance and guidelines, 2012). The collection of blood samples in newborn population poses additional challenges resulting in higher rates of contamination with growth of skin commensal organisms. As such, neonates who may not be infected often receive antibiotics inappropriately for a prolonged period of time. This has a negative impact on antimicrobial stewardship and may lead to adverse events and complications, including increased risk of death, necrotizing enterocolitis, late onset sepsis, alteration of gut colonization and increased risk of candida colonization and subsequent invasive candidiasis (Cotten et al., 2009; Esaiassen et al., 2017; Fjalstad et al., 2018).

**Figure 1:**
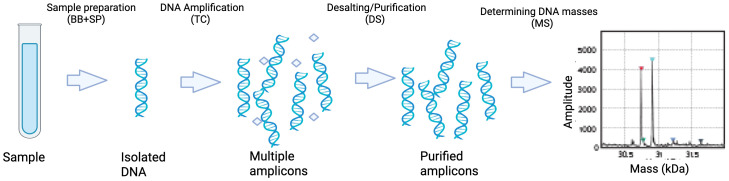
IRIDICA BAC BSI (PCR ESI-MS) assay.

Molecular methods have the potential to overcome many limitations of blood culture (Pammi et al., 2017). Multiplex PCR targeted against a limited number of known pathogens in neonatal population is one such option but has the disadvantage of not detecting unusual organisms. IRIDICA BAC BSI assay (IRIDICA^®^, Abbott Laboratories, Abbott Park, IL, USA) uses PCR for amplification of the 16S rRNA gene coupled with electrospray ionization-mass spectrometry (PCR/ESI-MS) to determine the molecular weight of the amplified products which is then compared it to a database of known molecular mass profiles. This enables rapid identification of more than 780 bacteria and candida in a sample within 6-8 hours whereas culture-based technology can take up to 48 hours (**Figure 1**) (Wolk et al., 2012).

There is a paucity of studies, however, taking this analysis further and exploring the clinical correlations and outcomes of the detection of bacterial DNA in blood in the neonatal cohort, with the view to develop future interventional studies.

The aim of this study was to assess the relationship between clinical features, routine laboratory parameters, including blood culture, and identification of micro-organisms by PCR/ESI-MS in infants with suspected early-onset infection.

## 2 Methods

### 2.1 Study design, population and ethics

We conducted a prospective observational cohort study at a neonatal intensive care unit and postnatal ward in a tertiary hospital within an urban setting. Neonates of gestational age of 34 weeks or more born at the Royal London Hospital with suspected early-onset infection were recruited prospectively from January 2016 till March 2017.

Parents were approached at the time of screening for infection before starting antibiotic treatment. The clinical evaluation of the newborn and initiation of antibiotic therapy was based on the presence of risk factors for and clinical indicators of EOS, as outlined in the NICE clinical guideline (Neonatal infection (early onset): antibiotics for prevention and treatment | Guidance and guidelines, 2012). The initial work up for suspected infection included blood sample collection for full blood count, C-reactive protein and blood culture. In addition to this an additional 0.5 ml was collected for identification of bacteria using IRIDICA. There is an absolute necessity to start antibiotic treatment promptly, and the micro-organism DNA might be affected if delayed sampling was undertaken. The parents were informed about additional blood sample collection and assent was obtained. After blood collection and administration of antibiotics, the parents were approached for full informed consent for the study. To facilitate the process of obtaining assent, parents of babies who are at risk of infection were given information before the birth of baby. The study and consent process was approved by NHS National Research Ethics Service (15/LO/1933).

### 2.2 Sample collection and processing

Blood samples were taken at the time of suspected infection by venepuncture for both blood culture inoculation (BacT/ALERT^®^ system) and PCR/ESI-MS analysis. The blood samples were collected by resident doctors who were trained for obtaining blood sampling in newborn infants. The blood samples were collected using aseptic techniques after preparing site of venepuncture with 2% chlorhexidine in 70% alcohol and personnel used gloves during collection. To avoid bacterial DNA contamination, blood culture and PCR/ESI-MS samples were collected using separate sterile needle and syringes. The blood samples for PCR/ESI-MS analysis were collected in DNA free sterile EDTA bottles. The blood samples volume of 0.5 ml was selected to allow bacterial identification with 97% specificity by PCR/ESI-MS (IRIDICA^®^) ((personal communication Oliver Kram, Paediatric Medicine, University Hospital of Geneva). The blood samples were processed with IRIDICA BAC BSI assay for PCR/ESI-MS using manufacturer’s instructions. The quality control measures were used to ensure assay accuracy. These included internal amplification controls to verify successful PCR amplification, nucleic acid free buffer for extraction control and a PCR/ESI-MS negative control which was processed in the same way as samples. Additionally, internal PCR and mass spectrometry controls were run with each sample to validate assay performance.

### 2.3 Data collection

An electronic database was used to document demographic and clinical details of each infant. The following baseline demographic data was collected: gestation, sex, birth weight, mode of delivery, duration of rupture of membranes.

Clinical and laboratory data were documented including: risk factors for early-onset neonatal infection (reference according to NICE CG149), clinical indicators of possible early-onset neonatal infection (observations and events in the baby) at time of diagnosis (reference according to NICE CG149), antibiotic choice and duration, inflammatory marker results in the first 72 hours (CRP, white blood cell count, platelet count) and chest radiograph findings (if completed). Finally, the clinical progress and outcome of each patient was also recorded.

### 2.4 Statistical analysis

Descriptive statistics were calculated for infant demographics and data was assessed for normality of distribution. As data was not normally distributed, Mann Whitney U test was used to compare demographic and laboratory parameters in infants with and without bacterial DNA detected in blood. Chi squared test or Fisher’s exact test was used to compare raised inflammatory markers in infants defined as CRP of > 5mg/ml with and without bacterial DNA detected in blood. All statistical analyses were completed using the statistical package SPSS (version 25) Differences were considered statistically significant when p values were < 0.05 in two tailed comparisons.

## 3 Results

Fifty-four infants born at >34 weeks’ gestational age were enrolled in the study.

Median (IQR) gestational age and birth weight were 39.7 (37.5-41.0) weeks and 3.2(2.7-3.5) kg respectively (**Table 1**). Median (IQR) duration of antibiotic administration was 2 (2–5) days. The actual blood samples volume received in the laboratory for PCR/ESI-MS analysis were between 0.25-0.5 mls.

**Table 1:** Demographics and laboratory parameters. Data is given as median (IQR).

	All infants (n=54)	PCR/ESI-MS positive for bacterial DNA (n = 10)	PCR/ESI-MS negative for bacterial DNA (n = 44)	p value*
Sex (M:F)	31:23	5:5	26:18	0.6
Gestational age (weeks)	39.7 (37.8 -41)	40.5 (39.5-41.2)	39.4 (37.1-40.9)	0.06
Birth weight (kg)	3.17 (2.69 – 3.54)	3.30 (3.07-3.63)	3.14 (2.62-3.53)	0.17
Rupture of membranes (hours)	13 (0–33)	29.5 (5.3-34.5)	10.5 (0-32.0)	0.19
Antibiotic duration (days)	2 (2–5)	3.5 (2–5)	2 (2–5)	0.32
Initial CRP (mg/L)	2.5 (2.5-2.5)	4.8 (2.5-12.3)	2.5 (2.5-2.5)	0.002
Repeat CRP at 18-24 hours(mg/L)	5 (2.5-23)	12 (5.8-52)	2.5 (2.5-18.3)	0.02
Max CRP in 1^st^ 72 hours (mg/L)	6.5(2.5-29)	12 (6.8-52)	2.5 (2.5-25.3)	0.03
Max WBC in 1^st^ 72 hours (10^9^/L)	16.4 (12.4-19.6)	19.2 (12.2-27.8)	16.0 (12.8-18.3)	0.29
Abnormal WBC (<5 or >30)	2	2	0	0.03
Platelets	213 (172-246)	225 (170–240)	212(171-248)	0.71

* denotes p value as per Mann Whitney U test.

Forty-three (80%) infants had one or more perinatal risk factors for early-onset infection (**Table 2A**). The most common risk factors were: premature rupture of membranes (n=36, 67%), parenteral antibiotics given to mother (22, 41%) and intrapartum fever or chorioamnionitis (n = 18, 33%). The common symptoms suggestive of infection in this cohort were: respiratory distress before 4 hours of age (n=21, 38.9%), hypoxia (n=12, 22.2%), jaundice within 24 hours of birth (n=8, 14.8%) and metabolic acidosis with base deficit of greater than 10 mmol/l (n=7, 13%). There were 36 (67%) infants with one or more symptoms of infection (**Table 2B**). All these infants survived to discharge.

**Table 2a:** Maternal, antenatal and perinatal risk factors for early onset sepsis.

	All infants (n=54)	Bacterial DNA detected by IRIDICA (n=10)	Bacterial DNA not detected by IRIDICA (n=44)	p value*
Risk factors	Yes n (%)	Yes n(%)	Yes n(%)	
Parenteral antibiotic treatment given to the woman for confirmed or suspected invasive bacterial infection (such as septicaemia) at any time during labour, or in the 24 hour period before or after birth.	22 (40.7)	5 (50)	17 (39%)	0.50
Suspected or confirmed infection in another baby in the case of a multiple pregnancy	0 (0)	0 (0)	0 (0)	
Invasive group B streptococcal infection in a previous baby	1 (1.9)	0 (0)	1 (2)	1.0
Maternal group B streptococcal colonization, bacteruria or infection in the current pregnancy	6 (11.1)	1 (10)	5 (11)	1.0
Premature rupture of membranes	36 (66.7)	8 (80)	28 (64)	0.32
Suspected or confirmed rupture of membranes for > 18 hours in a preterm birth	4 (7.4)	1 (10)	3 (7)	0.57
Preterm birth (<37 weeks) following spontaneous labour	8 (14.8)	0 (0)	8 (18)	0.32
Intrapartum fever higher than 38 °C, or confirmed or suspected chorioamnionitis	18 (33.3)	4 (40)	14 (32)	0.62
Any risk factor	43 (79.6)	8 (80)	35 (80)	1.0

* denotes p value as per Chi-square test or Fisher’s exact

**Table 2b:** Clinical symptoms and signs of early onset sepsis.

Clinical indicators	All infants (n=54)	Bacterial DNA detected by IRIDICA (n=10)	Bacterial DNA not detected by IRIDICA (n=44)	p value*
	Yes n (%)	Yes n(%)	Yes n(%)	
Respiratory distress starting> 4 hours after birth	2 (3.7)	0(0)	2(4.5)	1.0
Seizures	1 (1.9)	1 (10)	0 (0)	0.18
Need for mechanical ventilation in a term baby	3 (5.6)	0 (0)	3 (6.8)	1.0
Signs of shock	1 (1.9)	0 (0)	1 (2.3)	1.0
Feeding difficulties (for example, feed refusal)	0 (0)	0(0)	0(0)	
Feed intolerance, including vomiting, excessive gastric aspirates and abdominal distension	1 (1.9)	0(0)	1(2.3)	1.0
Signs of respiratory distress before 4h of age	21 (38.9)	5 (50)	16(36.4)	0.48
Apnoea	4 (7.4)	1 (10)	3(6.8)	0.57
Need for mechanical ventilation in a preterm baby	1 (1.9)	0 (0)	1(2.3)	1.0
Abnormal heart rate (bradycardia or tachycardia)	1 (1.9)	0 (0)	1(2.3)	1.0
Hypoxia (for example, central cyanosis or reduced oxygen saturation level)	12 (22.2)	2 (20)	10(22.7)	1.0
Need for cardio-pulmonary resuscitation	1 (1.9)	0 (0)	1(2.3)	1.0
Persistent foetal circulation (persistent pulmonary hypertension)	1 (1.9)	0 (0)	1(2.3)	1.0
Altered muscle tone (for example, floppiness)	2 (3.7)	1 (10)	1(2.3)	0.34
Altered behaviour or responsiveness	1 (1.9)	1 (10)	0 (0)	0.18
Signs of neonatal encephalopathy	1 (1.9)	1 (10)	0 (0)	0.18
Jaundice within 24 hours of birth	8 (14.8)	1 (10)	7(15.9)	1.0
Temperature abnormality (lower than 36C or higher than 38C) unexplained by environmental factors	3 (5.6)	0 (0)	3(6.8)	1.0
Unexplained excessive bleeding, thrombocytopaenia, or abnormal coagulation (INR > 2.0 in a term baby)	2 (3.7)	1(10)	1(2.3)	0.34
Oliguria persisting beyond 24 hours after birth	1 (1.9)	1 (10)	2(4.5)	0.46
Altered glucose homeostasis (hypoglycaemia or hyperglycaemia)	5 (9.3)	2 (20)	3(6.8)	0.22
Metabolic acidosis (base deficit of 10 mmol/litre or greater)	7 (13.0)	2 (20)	5(11.4)	0.60

* denotes p value as per Chi-square test or Fisher’s exact.

Only one infant in this cohort had a positive blood culture growth identified as Group B *Streptococcus*, whereas 10 infants had bacterial DNA identified by PCR/ESI-MS in blood (**Tables 3**, **4**). Bacterial DNA identified by PCR/ESI MS were; Group B *Streptococcus* 3, *Sneathia* 1 and *Cutibacterium acnes* (formerly *Propionibacterium acnes)* 6. All infants with no bacterial DNA detected on PCR/ESI-MS had a negative blood culture result. There was no significant difference in the risk factors and clinical features between the two infant groups (**Tables 2A**, **2B**). Infants with bacterial DNA detected by IRIDICA had higher initial C-reactive protein levels and higher CRP levels at 18-24 hours after birth and higher maximum CRP (**Table 1**). Of the 29 neonates with raised maximum CRP of >5 mg/L, 9 (31%, 95% CI 17-49%) had bacterial DNA detected in the blood sample while only one had bacterial growth on blood culture. The 25 neonates with persistently low CRP (<5 mg/L), 24 (96%, 95% CI 83-99%) had no bacteria detected in the blood using PCR/ESI MS (**Table 5**). On the other hand, 20 infants with no bacterial DNA detected using PCR/ESI-MS 20 had raised CRP of >5 mg/L giving a negative predictive value of 54% (**Table 5**). Newborns with CRP >5mg/L had a significantly higher odds of having bacterial DNA detected in blood in comparison with those with maximum CRP of <5 mg/L (OR 10.8, 95% CI 1.3 – 92.6). The detection of bacterial DNA by PCR/ES-MS had a sensitivity of 100%, specificity of 83% and negative predictive value of 100% in comparison with positive blood culture in this cohort (**Table 4**).

**Table 3:** Clinical details of babies with bacterial DNA detected in blood samples using PCR/ESI MS (IRIDICA).

Gestational Age (weeks)	Birth weight (gm)	Any risk factor for sepsis	Bacterial DNA detection by IRIDICA	Blood culture	Clinical details	Max CRP (mg/L)	Max WBC (x10^3^/ L)	Antibiotics duration (days)
40.1	2740	Yes	*Streptococcus agalactiae*	*Strepto- coccus agalactiae*	Emergency CS for pathological CTG. Born in poor condition. Developed respiratory distress. GBS +ve from gastric aspirate sample	51	21.6	7
40.6	3400	Yes	*Streptococcus* sp*ecies, eikenella corrodens, streptococcus infantis/peroris*	No growth	SVD, Maternal thrombocytopenia, GBS colonization.	21	18.7	5
41.6	2990	Yes	*Streptococcus agalactiae/Enteroc -ooccus cecorum*	No growth	KIWI delivery for fetal bradycardia, born in poor condition with meconium stained liquor, respiratory distress, lactic acidosis, ear swab GBS +ve	55	11.5	5
41.6	3999	Yes	*Sneathia* sp*ecies*	No growth	Emergency CS for pathological CTG, maternal chorioamnionitis, Hypoxic ischaemic encephalopathy	173	10.5	7
40.4	3750	Yes	*Cutibacterium acnes*	No growth	Emergency CS for fetal tachycardia, PROM 28 hours, maternal pyrexia on IV antibiotics, clinically well	11	30.9	5
39.0	3200	Yes	*Cutibacterium acnes*	No growth	SVD, Poor respiratory effort at birth requiring resuscitation, PROM 31 hours, clinically well	13	12.4	2
39.7	3090	No	*Cutibacterium acnes*	No growth	SVD, Haemolytic jaundice secondary to ABO incompatibility, serum bilirubin at exchange transfusion level, phototherapy given	7	26.8	2
39.0	3180	Yes	*Cutibacterium acnes*	No growth	SVD, maternal pyrexia on IV antibiotics, Left shifted neutrophil and toxic granulations	<5	19.6	2
41.0	3540	Yes	*Cutibacterium acnes*	No growth	SVD, maternal pyrexia on IV antibiotics, PROM 41 hours, Poor feeding due to tongue tie	9	12.4	2
41.1	3585	Yes	*Cutibacterium acnes*	No growth	Emergency CS for PROM 32 hours and meconium stained liquor, Received resuscitation, CPAP and oxygen. Admitted to neonatal unit on CPAP, right sided pneumothorax, E coli grown from Gastric aspirate sample	6	38.6	2

SVD, spontaneous vaginal delivery; CS, Caesarean section; CTG, cardiotocogram; GBS, Group B Streptococcus; PROM, prolonged rupture of membranes.

**Table 4:** Cross-tabulation of positive blood culture and bacterial detection by PCR/ESI-MS. Diagnostic test values of PCR/ESI-MS for positive blood culture.

		Blood culture			Diagnostic test values	
		Positive (n)	Negative (n)	Total number	Sensitivity	100%
PCR/ESIMS	bacterial DNA detected (n)	1	9	10	Specificity	83%
	bacterial DNA not detected (n)	0	44	44	PPV	10%
Total		1	53	54	NPV	100%

PPV, Positive predictive value; NPV, Negative predictive value.

**Table 5:** Cross-tabulation of maximum CRP levels and bacterial detection by PCR/ESI-MS. Diagnostic test values of PCR/ESI-MS for raised CRP.

		CRP			Diagnostic test values	
		≥5 mg/L (n)	<5 mg/L (n)	Total number	Sensitivity	31%
PCR/ESIMS	bacterial DNA detected (n)	9	1	10	Specificity	96%
	bacterial DNA not detected (n)	20	24	44	PPV	90%
Total		29	25	54	NPV	54%

Fisher’s Exact test p=0.014.

The proportion of infants with a CRP value ≥ 5 was significantly higher if bacterial DNA had been detected (p=0.014).

Of the 6 infants with *C. acnes* detected by PCR/ESI-MS, 3 were born to mothers with pyrexia receiving intravenous antibiotics, 4 had PROM ranging from 28-41 hours, 2 required resuscitation at birth, 5 had raised CRP with the maximum CRP levels of these infants were between 6-13 mg/L and 3 had abnormal white blood cell count (**Table 3**).

## 4 Discussion

PCR/ESI-MS was four times more likely to identify an organism that is known to be pathogenic in the neonatal period than standard culture; and identified presence of *C. acnes* DNA in blood in a further 6 cases. *C. acnes* and *Sneathia*, which are known skin or vaginal commensals; are often difficult to culture in traditional blood culture methods. This was similar to the experience from critically ill adults in the RADICAL study where the PCR/ESI-MS based technique resulted in 3 fold greater number of bacterial identification compared to culture (Vincent et al., 2015). Of note, the detection of microbial DNA but not cultured bacteria was associated with increased mortality in patients with suspected sepsis in the RADICAL study (O’Dwyer et al., 2017). The PCR/ESI-MS based technique in our study has shown high sensitivity and negative predictive value. This is concordant with experience of using this technique in adults receiving critical care (Vincent et al., 2015; O’Dwyer et al., 2017; Strålin et al., 2020). To our knowledge this is first study reporting the utility of PCR/ESI-MS specifically on term and near term newborn with suspected or confirmed early onset sepsis.

### 4.1 Comparison with other studies in newborn infants using PCR/ESI-MS

Only one previous study has examined this molecular method in the newborn cohort. Delco et al. conducted a prospective cohort study on 114 neonates including preterm infants to determine the efficacy of PCR/ESI-MS as a diagnostic tool compared to conventional blood cultures in for both EOS and LOS (Delco et al., 2017). They reported a sensitivity and specificity of 80 and 84% respectively and a high negative predictive value (98%) when compared to blood cultures as gold standard (Delco et al., 2017). Our data is in keeping with the study by Delco et al. with a high sensitivity of 100%, specificity of 83% and negative predictive value of 100%.

### 4.2 Comparison with studies on infants with EOS using other PCR techniques

Laforgia et al. reported bacterial DNA detection using broad range conventional PCR amplifying conserved 16s rRNA region but bacterial identification was not possible (Laforgia et al., 1997). All infants with positive blood culture had bacterial DNA detected (4 out of 33) and there were 2 further infants with positive PCR but negative blood cultures. Another study using similar methods reported a specificity of 97.5% compared to blood culture in newborn with suspected EOS, but raised concerns as PCR failed to detect 10 out of 17 positive blood cultures(sensitivity 41%) (Jordan et al., 2006). In contrast, the PCR/ESI-MS based technology in our study had a sensitivity of 100% and a specificity of 83%.

A commercial multiplex PCR SeptiFast^®^-test (LightCycler^®^ Roche Diagnostics, Mannheim, Germany) that detected more than 20 pathogens by targeting the internal transcribed sequences situated between 16S and 23S bacterial ribosomal RNA as well as between 18S and 5.6S fungal ribosomal RNA has been used for evaluating LOS (Tröger et al., 2016; Straub et al., 2017) and recently for EOS (Stein et al., 2023). Multiplex-PCR was negative in 10 episodes out of 55 episodes with positive blood culture in LOS (Tröger et al., 2016). In this group, only one case with coagulase-negative staphylocci (CoNS)-positive blood culture and negative multiplex-PCR was classified as `no infection`and CoNS was considered as a contaminant. All other blood culture results represented clinically significant infections. In the study by Straub et al, the PCR was negative in 5 out of 51 cases with positive blood culture and was positive in 23 of 85 infants with no sepsis (Straub et al., 2017). In the cases of early onset neonatal sepsis, the SeptiFast test failed to detect pathogens in the two instances of proven blood culture sepsis (Stein et al., 2023). Multiplex PCR testing identified pathogens in 19 cases from patients in suspected early onset neonatal sepsis but only two were deemed potentially true positives, and both fell under the EOS category “sepsis likely”.

Another study evaluating multiplex PCR targeting the eight most common bacteria in preterm infants with suspected or confirmed LOS reported no significant difference in detection rate of sepsis in comparison with blood cultures (van den Brand et al., 2018). Oeser et al. have reported an evaluation of babies with EOS with multiplex PCR targeting 6 bacteria (Oeser et al., 2020). They found *Staphyloccous aureus*, *Enterobacteriaceae* and *Streptococcus pneumoniae* in 24%, 20% and 18% of samples respectively, while Group B *Streptococcus* was isolated in 15% of samples only. Some of these organisms are not fastidious to grow and patterns and prevalence of causative organism for EOS is unusual in this study. While our study using PCR/ESI-MS which can detect up to 800 bacterial species identified bacteria in 18.5% of blood samples including Group B *Streptococcus* in 6% of blood samples. They did not find an association between bacterial detection by multiplex PCR and CRP technique, whereas we found a strong correlation between bacterial DNA detection in blood by PCR/ESI-MS and CRP which may suggest a causal association. In comparison with multiplex PCR, the PCR ESI-MS uses a database of approximately 800 bacteria, fungi and DNA viruses allowing the detection of a very diverse range of organisms.

### 4.3 Identification of bacteria with fastidious growth requirements

Three types of organisms were identified in our study, *Streptococcus* sp., *C.acnes* and *Sneathia*, which are known skin or vaginal commensals; the latter two are often difficult to culture and, as such, molecular diagnostic techniques are often more useful. Of the streptococcal species, the pathogenicity of *Streptococcus agalactiae* (also known as *group B streptococcus* or GBS) in newborns and its association with increased neonatal morbidity and mortality is particularly well established. *Sneathia sanguinegens* (previously named *leptotrichia sanguinegens*) is a less well known fastidious Gram-negative anaerobe which commonly inhabits the female genital tract and has also been identified in amniotic fluid samples (DiGiulio et al., 2008; Harwich et al., 2012). Previous studies have linked vaginal *Sneathia* colonization to various adverse pregnancy outcomes, including preterm premature rupture of membranes, funisitis, chorioamnionitis, post-partum maternal fever as well as neonatal bacteraemia, EOS and meningitis (Hanff et al., 1995; Han et al., 2009; Devi et al., 2014; Brown et al., 2018). It is difficult to isolate using standard anaerobic cultures and, therefore, second line molecular techniques such as broad range conventional PCR assays targeting conserved 16S rRNA gene sequencing are often required to identify it within blood and CSF samples (Hanff et al., 1995; Devi et al., 2014). Our study similarly highlights its pathogenicity, given that the single patient with *Sneathia* detected had a poor clinical course and marked rise in inflammatory markers.

*Cutibacterium* is a micro aerophilic anaerobic bacterium that has fastidious growth requirements and is difficult to grow using standard cultures. Previous studies have isolated it from the surface of the skin, umbilical cord or conjunctiva in healthy neonates born by Caesarean section (Eder et al., 2005; Jimenez et al., 2005; Dominguez-Bello et al., 2010). Its pathogenic role, however, is less apparent. In the past it has been commonly regarded as contaminant (Hossain et al., 2016), however there is increased interest in its role in a range of conditions ranging from joint infections to prostate cancer. *Cutibacterium* has been detected by broad range conventional 16S rRNA PCR assay but not by blood culture in 2 of the 17 PCR positive blood specimens collected from 172 infants (Shang et al., 2005) and 3 out of 108 samples (Oeser et al., 2020). Another study by Brook et al. analyzed samples following needle aspiration of infected cephalhaematomas from six neonates between 1975 and 2003.

*C. acnes* along with *Peptostreptococcus magnus* was identified in the aspirate of one infant although there was no growth on blood culture and the patient had a good clinical outcome (Brook, 2005). Similarly, in our cohort, the infants whose blood samples were positive for *C. acnes* remained well, initiation of antibiotics was primarily for risk factors, CRP was only mildly elevated and most were only given 2 days of antibiotics therapy.

Cutibacterium has been detected in the placenta and amniotic fluid (Collado et al., 2016), although the recent reports contradict this findings and suggest that this may be a contaminant and been acquired during birth process (Gschwind et al., 2020; Panzer et al., 2023). The threshold for detection of *C.acnes* was set by the manufacturer’s at a higher level than that of conventional pathogens such as *E.coli* to reduce identification of low level contamination in this study, in addition the sample volume was a tenth of that used for adult detection of BSI, but it is still possible that the *C.acnes* DNA found in this study are contaminant from the babies skin and not from blood.

### 4.4 Implications of bacterial DNA detection in blood samples

It is not known if bacterial DNA detection in blood samples has similar implications as positive blood culture sepsis in newborn infants. While the positive blood culture represents presence of live bacteria in the blood, the presence of bacterial DNA might represent presence of already dead bacteria with free floating DNA or bacterial DNA engulfed by white blood cells. Interestingly in adults receiving critical care presence of bacterial DNA in blood but not a positive blood culture was associated with increased mortality (O’Dwyer et al., 2017). Even when no bacterial DNA was detected by PCR/ESI-MS, 20 out of 44 infants had raised inflammatory markers. We can postulate that this could be due to inflammatory response due to bacterial infection in other mucosal surfaces or organs rather than blood stream infection, nonbacterial pathogen or non-infective causes of inflammation.

### 4.5 Strengths of this study

This study was based on clinical and laboratory assessment using a more objective definition of suspected EOS with standardized criteria as outlined by national guidelines (Neonatal infection (early onset): antibiotics for prevention and treatment | Guidance and guidelines, 2012) which is in contrast with a self-reported limitation of the Delco et al. study. We primarily focused on CRP rise as a marker of infectivity in an attempt to elucidate if the organisms identified were causing systemic infection but not necessarily a blood stream infection. This is particularly helpful in the context of exposure of newborns to vaginal or skin commensals during birth process. This study demonstrates the potential benefit of PCR/ESI-MS in neonatal sepsis as a useful discriminator for ruling out EOS and therefore has a role to play in antimicrobial stewardship.

### 4.6 Weaknesses of this study

This small cohort of infants treated based on risk factors had a low rate of blood culture positivity which is consistent with clinical experience and could result from use of antibiotics in mothers. We have used smaller blood volumes (0.25-0.5 ml) than recommended by the manufacturer for the detection of blood stream infections in adults. This was based on the Delco et al. study which used 0.2 - 0.5mls and is appropriate from samples in the newborn. The use of 5mls of blood volume for bacterial DNA detection has been shown to have high specificity and negative predictive value in a study involving adults receiving critical care (Vincent et al., 2015; O’Dwyer et al., 2017). We have reported high rates of detection of bacterial DNA from *C. acnes* which is typically considered to be a commensal and its causative role in EOS in newborn is uncertain. The high rates of potential contaminant detection from blood samples despite having trained staff using a standardized guideline underscores the challenges and difficulties in obtaining samples in emergency situation in newborn infants. Theoretically, it may be possible to explore this further by recruiting healthy newborns without any risk factors for EOS, but it would be difficult to get ethical approval for blood sampling in this group of infants.

The use of PCR ESI-MS for suspected EOS in newborn allowed detection of bacterial DNA in blood samples with high sensitivity and negative predictive value. This would be useful as a ‘rule out’ test in infants with suspected early onset sepsis and has the potential to shorten antibiotics usage and improve antibiotics stewardship. This method can identify pathogens unrecognized by blood culture (*Sneathia* and possibly another *Streptococcus* spp.) but also has the potentially confounding influence of sampling difficulties. While quality improvement measures for obtaining blood culture methods have resulted in lower rates of skin commensal contamination in positive blood cultures, the use of molecular methods would require improvement in sampling methods to reduce the DNA contamination from organisms on skin surface.

Production of this system using PCR ESI-MS by IRIDICA assay has been discontinued (Tkadlec et al., 2020) but future development of the PCR ESI-MS technique and other molecular techniques such as use of metagenomics for identification of a broad range of bacterial DNA has the potential to improve diagnosis of sepsis in newborn (Alcolea-Medina et al., 2024). Tests to ‘rule out infection’ are useful, but with the burgeoning issues with antibiotic resistance, confidence in the significance of the findings with low false positive rates is also very important. While newer molecular methods with higher sensitivity for detecting infections are being developed, it is crucial that sample collections methods to improve contamination rates are also developed and assessed. The results are promising but there is a need to improve blood collection methods to take advantage of the potential benefits of molecular detection. The newer methods will require rigorous evaluation in neonatal populations because of unique challenges in obtaining samples and the higher risk of contamination with skin commensals leading to false positive results.

## 5 Conclusions

PCR/ESI-MS based technique for detecting bacterial DNA in blood samples has a high sensitivity and negative predictive value for identification of bacterial organism in term and near term newborn infants screened for early onset sepsis. It was able to detect organisms considered pathogenic in four times more blood samples than blood culture alone. Bacterial DNA was detected by PCR/ESI-MS in infants who did not have bacterial growth on blood culture and this was associated with a raised inflammatory marker. It may be a useful tool to exclude sepsis in the neonatal cohort and reassess the need for prolonged antibiotic treatment. The results are promising but there is a need to improve blood collection methods to take advantage of the potential benefits of newer molecular methods with higher sensitivity to detect infections. The newer methods should be evaluated critically in newborn population because of inherent challenges.

## Data Availability

The datasets presented in this article are not readily available because there is no generated dataset in this study. The real data has been analyzed and summarized. Requests to access the datasets should be directed to ajay.sinha@nhs.net.
